# Assessment of Chronic Multi-Electrode Spinal Cord Electrical Stimulation and Electromyography Platform in Non-Human Primates

**DOI:** 10.3390/biomedicines14010166

**Published:** 2026-01-13

**Authors:** Alena D. Militskova, Vyacheslav. V. Andrianov, Artur R. Biktimirov, Evgeny. V. Gulaev, Tatiana. B. Alfimova, Matthew. O. Shkap, Larisa A. Burachek, Roman V. Panfilov, Dmitry. V. Bulgin, Sergey. V. Zhirnov, Alexander. P. Trashkov, Igor A. Lavrov, Vladimir P. Baklaushev

**Affiliations:** 1 Federal Center of Brain Research and Neurotechnologies, Federal Medical Biological Agency of Russia, Moscow 117513, Russia; mamashotmilktea@gmail.com (A.D.M.); vvandrianov@kpfu.ru (V.V.A.); biartur2006@yandex.ru (A.R.B.); evlgul@mail.ru (E.V.G.); doctor168611@gmail.com (T.B.A.); shkap010@mail.ru (M.O.S.); 2 Institute of Fundamental Medicine and Biology, Kazan Federal University, Kazan 420008, Russia; 3 National Research Center “Kurchatov Institute”, Moscow 123098, Russia; larisa-burahek88@mail.ru (L.A.B.); zxcdsa-1@mail.ru (R.V.P.); molmed1999@yahoo.com (D.V.B.); trashkov_ap@nrcki.ru (A.P.T.); 4 Institute of Biomedical Engineering, National University of Science and Technology MISIS, Moscow 119049, Russia; parhomenko.dot@gmail.com; 5 Department of Biomedical Engineering, Mayo Clinic, Rochester, MN 55905, USA; lavrov.igor@mayo.edu; 6 Department of Medicinal Nanotechnologies, Pirogov Russian National Research Medical University, Ministry of Health of the Russian Federation, Moscow 117997, Russia; 7 Laboratory of Regenerative Medicine, Research Institute of Pulmonology, Federal Medical Biological Agency of Russia, Moscow 115682, Russia

**Keywords:** spinal cord injury, SCI, *Macaca mulatta*, monkey, rhesus macaques, epidural electrical stimulation, neuromodulation, motor-evoked potentials, kinematics

## Abstract

**Background/Objectives**: Traumatic spinal cord (SC) injury (SCI) is a debilitating neurological condition. Minimally invasive approaches to monitor in real time the functional state of the neuromotor apparatus in animal models of SCI (at rest and movement) to assess effectiveness of therapy are needed in preclinical studies. We aimed to develop such a bioethically acceptable platform for SCI studies on non-human primates (Rhesus macaques). **Methods**: Epidural and myographic electrode implantation (EI) (wireless and wired, connected via a head plug) was performed. After EI, motor responses caused by electrical stimulation of the SC at the level of the cervical and lumbar thickening were recorded; electromyography of the limb muscles was recorded during quadrupedal movement of the animal on a treadmill with simultaneous assessment of movements’ kinematic parameters. Five weeks after EI, three animals underwent lateral hemisection of the SC in the C4–C5 segment under the control of a surgical microscope and intraoperative recording of motor- and sensory-evoked potentials. **Results**: Within 30 days after SCI, during treadmill testing, a decrease in electromyographic activity of the limb muscles and the volume of angular movement in the joints on the side of the injury was detected. Electrical stimulation at the L2–S1 segments of the SC at a frequency of 30 Hz led to the appearance of a locomotor pattern in the muscles of the hind limbs and an increase in the range of motion. **Conclusions**: Our platform can be used for pathophysiological studies of various neuromodulation modes and as a basis for the development of control neurointerfaces.

## 1. Introduction

Spinal cord (SC) injury (SCI) is a debilitating condition that leads to severe neurological disorders [1]; worldwide, between 250,000 and 500,000 people suffer SCI every year [2]. The pathophysiological mechanisms which are triggered after SCI are complex [1]. The spectrum of functional disorders caused by SCI may result not only from direct traumatic damage to the SC, but also as a consequence of subsequent ascending and descending neurodegeneration, deafferentation, loss of voluntary control, cerebrospinal fluid circulation disorders, etc. [3,4,5,6,7]. Due to the insufficient effectiveness of existing treatment methods and the extremely high disability impact of SCI, many regenerative technologies are being developed as potential curative approaches to SCI, requiring adequate preclinical testing [8].

Epidural electrical stimulation (EES) of the SC is a well-established method of neuromodulation that is currently used in clinical practice to alleviate the effects of SCI: it is used for alleviating chronic pain [9], for improvement of non-motor functions [10] and in studies focusing on the rehabilitation of the musculoskeletal system after SCI, both in animals [11,12,13,14] and in humans [15,16,17]. The effectiveness of EES in restoring lost functions after SCI is due to stimulation of the posterior roots in the lumbar thickening of the SCI and activation of the gait generator [16,17,18,19]. In addition, in recent years, the field of research on animal models combining SC electrical stimulation with other therapeutic interventions has been expanding [20]. However, despite significant advances in EES research, uncertainty remains regarding the transferability of these results to clinical practice.

Rodent models have become widely used in biomedical research aimed at studying pathophysiological processes [21]. For example, it has been shown in rats that EES of the lumbar spinal cord can lead to the activation of synergistic muscle groups, providing locomotion close to natural [22,23]. In this experimental model, electrical stimulation of the spinal cord is usually performed using an external stimulator attached via a special connector on the animal’s head (“head plug”), and electrodes in the form of wires are connected to the lumbar region of the spinal cord [24,25,26,27]. Using a rodent model of SCI, significant progress has been made in understanding the basic mechanisms of plasticity in the SC modulated through EES [25].

To translate the latest advances in neuromodulation and gene–cell technologies into clinical medicine, testing novel therapies on large mammals, most relevantly non-human primates (NHPs), is a necessary link between the rat model and humans. The SC of NHPs is anatomically and physiologically similar to the human SC, especially in terms of the topography of the conduction pathways and the function of the corticospinal tracts [28]. Without experimental research on primates, it is impossible to develop neurotechnology involving the implantation of invasive cortical electrodes and the creation of control neurointerfaces. At the same time, when modeling SC pathology in NHPs and developing methods of therapy, the high level of development of higher nervous activity in these animals should be taken into account. Therefore, minimally invasive methods of implantation of epidural and myographic electrodes are used to assess the neurophysiological and locomotor consequences of SCI and the effectiveness of the methods used to treat it. While rodent models currently dominate the research landscape for EES after SCI, NHP models are essential for clinical translation due to their unique neuroanatomical and physiological similarities to humans. However, there is a significant lack of reliable, fully integrated platforms that enable synchronized, chronic recording of EMG and kinematics in freely moving primates following SCI.

The aim of this study was to develop a bioethically acceptable platform for research on NHP using minimally invasive methods of implantation of epidural and myographic electrodes (both wireless and wired, connected via a head plug) to assess the neurophysiological and locomotor consequences of SCI and the effectiveness of the methods used to treat it.

## 2. Materials and Methods

### 2.1. Animals and Care

All experiments on NHP were conducted at Kurchatov Medical Primatology Center in Sochi, Russia. The animals used for the study were selected from 20 male rhesus macaques aged 2–4 years and weighing up to 5 kg, which had been previously removed from the enclosure and placed in cages. The description of the animal selection process was categorized based on three selection stages (Supplementary Figure S1): Clinical screening, behavioral habituation, and treadmill skill. Of these 20 animals, the 5 most suitable for the study, which showed the best quadrupedal walking skills on the treadmill, were selected. The selected animals were kept in conditions of natural lighting (at least 12 h per day) with free access to water and were fed twice daily in spacious cages with enrichment items. All manipulations with animals were carried out in accordance with the European Convention for the Protection of Vertebrate Animals used for Experimental and other Scientific Purposes (European Treaty Series—No. 123, Strasbourg, 18 March 1986; Directive 2010/63/EU of the European Parliament and of the Council of 22 September 2010 on the protection of animals used for scientific purposes). The experimental protocol was approved by the Local Ethics Committee of the NRC Kurchatov Institute (Protocol No. 02-2 from 20 March 2025). The monkeys developed the necessary motor skills in their lower and upper limbs, after which electrodes were implanted and spinal trauma was simulated. During the experiment, two animals were excluded from the study due to failure of the implanted devices. As a result, three animals underwent a complete neurophysiological examination.

### 2.2. Electromyography Wire and Electrode Implants

**Figure 1:**
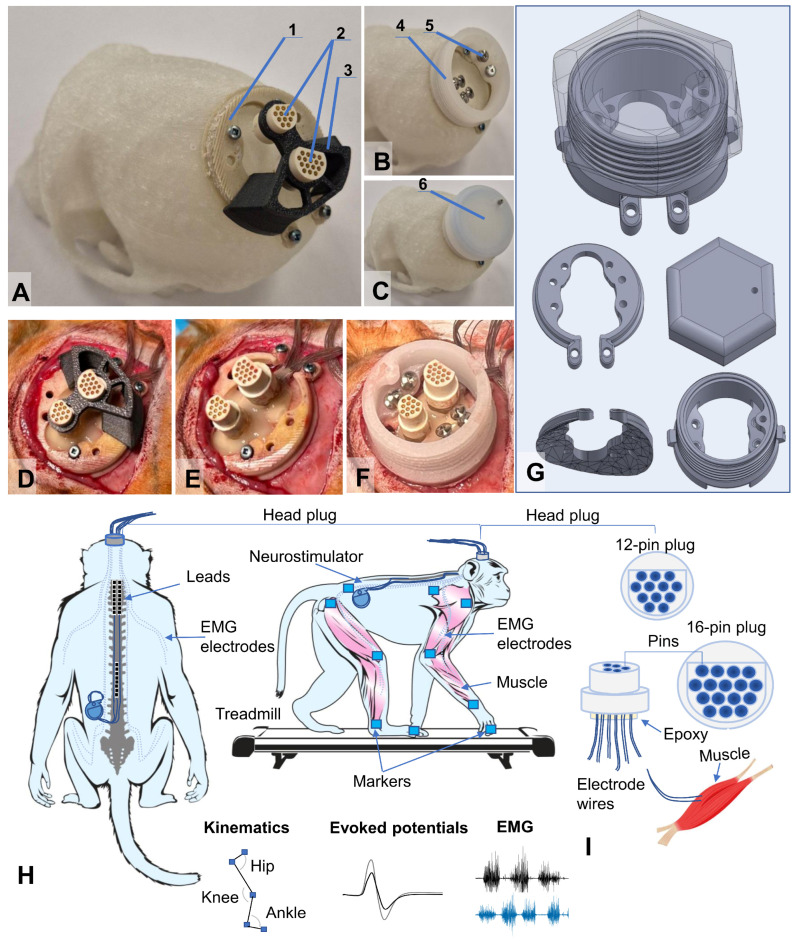
Head plug design and general diagram of implantable devices. (**A**–**C**) Head plug prototype installed on an individual 3D model of a rhesus macaque skull, which was subsequently implanted. **Designations:** (1) substrate made of a composite biocompatible and osseointegrative material based on polyhydroxybutyrate and hydroxyapatite particles; (2) 12- and 16-pin plugs; (3) liner for positioning the plugs after installing the substrate—until fixation with light-curing composite; (4) head plug body with thread for the cover, made of polypropylene; (5) self-tapping screws made of titanium alloy for fixing the structure to the skull; (6) sealed cover with thread and locking screw; (**D**–**F**) stages of head plug installation during surgery on a rhesus macaque; (**G**) 3D models of head plug elements in a customized design; (**H**) general diagram of the implantable system for simultaneous recording of motor activity and spinal cord stimulation; (**I**) diagram of connectors for head plugs.

We developed an implantable system for simultaneous recording of motor activity and spinal cord stimulation. To connect the EMG equipment and stimulators to the implantable electrodes, head plugs were manufactured, including connectors (one 12-pin and one 16-pin, A22004-001 (MCP-12-SS), and A22032-001 (MCP-16-SS), Omnetics Connector Corp., Minneapolis, MN, USA) and microcables in multi-strand steel insulation (AS632, Cooner Wire, Chatsworth, CA, USA) as electrical conductors (Figure 1A–C).

The lengths of the wires for implantation were calculated based on measurements of the research objects. The calculation of the lengths of the wires for implantation was performed using a multi-stage scheme, taking into account the dimensions of the research object.

The design of the connectors attached to the skull of the research object included a box with a hermetically sealed space, fixed to the bone tissue using a light-curing biocompatible dental composite. This head plug design allows microcontacts to remain in working order and ensures easy connection to the mating parts of these connectors for connection to EMG recording equipment and stimulators. As a result of initial tests of the box for plugs attached to the bones of the cranial vault, it was decided to develop a second generation of the box made of osteoinductive material, which ensured reliable fixation of the box to the cranial vault with bone ingrowth (Figure 1C).

The implantation kit consisted of two parts: insulated electrical wires soldered to a connector (plug) and a container. The wires were connected to the plug using low-temperature solder (POS-61), followed by insulation of the solder joints with epoxy glue. During implantation, the plug itself, as well as the part covered with glue, was insulated from biological tissues by the container material, the outer part of which was made of a biocompatible composite. The material of the implanted wires (Hookup Wire, Stranded Stainless Steel, model AS 632, Cooner Wire Company, Chatsworth, CA, USA) was a stainless-steel core insulated with fluorinated ethylene propylene (FEP). The wire was made in the form of a single-core twist based on ultra-thin filaments (15 pcs) in a common outer insulation. The outer diameter of the wire in insulation was 0.3 mm, and the impedance was 1 Ohm/cm. The kit, including wires and contact plugs, was manufactured in the Neuroregeneration laboratory of the Federal Medical Biological Agency of Russia. The osteoinductive head plug was developed and manufactured at the Institute of Biomedical Engineering, National University of Science and Technology MISIS (patent RU 2 829 633 C1, 26 June 2024, https://www.fips.ru/cdfi/fips.dll/ru?ty=29&docid=2829633, accessed on 7 January 2026).

### 2.3. Electrode Implantation

The electrode implantation technique was adapted for primates based on a previously used implantation technique in rodents [24,25,29,30]. After making an incision in the skin in the projection of the parietal bone perpendicular to the midline suture of the skull, the muscles and fascia were retracted laterally, and the skull was thoroughly cleaned and dried. In several stages, a head plug was installed on the skull according to the developed installation technology (Figure 1) using self-tapping screws (Conmet, Moscow, Russia) and medium-flow light-curing dental composite. Through additional incisions on the neck and back, wires were distributed subcutaneously to the corresponding muscles using special introducers. Incisions were made in the skin in the projection of the muscles under study, and in the fascial sheaths of m. biceps brachii (BIC), m. triceps brachii (TRIC), m. flexor digitorum superficialis (FLE), m. extensor carpi ulnaris (EXT), m. rectus femoris (RF), m. biceps femoris (BF), m. tibialis anterior (TA), and m. gastrocnemius medianus (GM). Bipolar intramuscular EMG electrodes were inserted into the muscles through small incisions in the fasciae and fixed with polyfilament non-absorbable sutures using the technique described earlier [26]. All EMG wires had additional length, from which subcutaneous stress-relief loops were formed and laid around each implantation site to avoid mechanical tension/stress on the wires during the animal’s movements and growth. To verify the correct placement of the electrodes in each muscle, electrical stimulation was performed through the head plug with visual control of muscle contraction and impedance recording. Test electrical stimulation was performed using a NeoStim 5 multichannel stimulator (KOSIMA, Moscow, Russia; website: https://cosyma.pro/ (accessed on 7 January 2026)), connected to the head plug via wired connections.

For epidural stimulation of animals, we used rod electrodes and implantable Precision Spectra generators (Boston Scientific, Marlborough, MA, USA). With the animal in the prone position, a linear incision was made along the midline of the lumbar spine in the S1–L4 projection. Under X-ray guidance, the epidural space was punctured with a Tuohy needle, and two cylindrical electrodes were sequentially inserted into the posterior epidural space and placed in the projection of the DREZ zone at the level of the C2–C7 vertebrae on the left and right sides (Figure 1H). A third cylindrical electrode was placed in the posterior epidural space along the midline at the level of Th10-L2. The needle was removed, and the electrodes were fixed to the aponeurosis. A subaponeurotic pocket was formed for the pulse generator on the left. The permanent neurostimulation system was mounted, and the generator was placed in the pocket and fixed with knot sutures. The electrode loops were placed and additionally fixed with threads to the aponeurosis. To record artifacts in the electrode projection, two wires with the Teflon coating removed to expose 1.0–1.5 mm of wire were inserted under the skin. Another wire with an exposed distal end (about 1 cm) was placed circularly subcutaneously in the lumbar region and served as a common ground [25].

### 2.4. Spinal Cord Injury

To avoid aspiration complications, food was discontinued at least 12 h before induction of anesthesia, and drinking was discontinued 4 h before. To ensure neuroleptanalgesia, for the safe removal of animals from the enclosure and further preoperative preparation, Relanium (1 mg/kg) and Ketamine (10 mg/kg) were administered intramuscularly into the quadriceps muscle of the thigh; once the desired anesthetic effect was achieved (5–10 min), with spontaneous, adequate breathing maintained, the animal was removed from the enclosure and transported to the preoperative room. A veterinary clipper was used to remove the hair on the back and limbs. Under aseptic conditions, a 20 G catheter was inserted into the subcutaneous vein of the lower leg. For the purpose of desensitization, multimodal analgesia, and achieving adequate perioperative hemostasis, 20 min before surgery, Ketonal 10 mg/kg, Dexamethasone 1 mg/kg, and Tranexamic acid 20 mg/kg were administered 20 min before surgery. Rapid sequential induction of general anesthesia was performed against a background of preliminary oxygenation and administration of Fentanyl 25 mcg/kg, Propofol 5–10 mg/kg, and Rocuronium 2.5–3 mg/kg. Once an adequate depth of anesthesia was achieved, orotracheal intubation was performed with a 4.0 tube, after which the animal was transferred to mechanical ventilation in a semi-closed circuit with the following parameters: tidal volume of 120–150 mL, respiratory rate of 23–26 per minute, FiO2 of 0.4. Anesthesia was maintained by bolus administration of Fentanyl and inhalation of isoflurane 1.2–2.0 vol%.

The surgical procedure was performed in a sterile operating room. Throughout the operation, the animals received infusion therapy with balanced crystalloid solutions at a rate of 10–15 mL/kg/h, and their heart rate, blood pressure, pulse oximetry, and capnography were monitored. The monkeys were placed in the prone position, and their skin was wiped with Povidone-iodine. A 5 cm longitudinal midline skin incision was made in the projection of the spinous processes of the C3–C5 vertebrae, and the paraspinal muscles were separated subperiosteally. The spinous process of C4 was removed, and interlaminectomy was performed with partial removal of the C4–C5 vertebrae. The dura mater was exposed and longitudinally dissected in the projection of the C4–C5 segment. Using a scalpel and microsurgical scissors under the control of an operating microscope and intraoperative recording of SSEP and MEP, a wedge-shaped hemisection of the spinal cord was performed on the right side. Upon receiving neurophysiological confirmation of the complete absence of afferent and efferent responses from the right half of the body, a hemostatic sponge was placed in the formed defect (2–3 mm) of the spinal cord. The DMSA was sutured and additionally sealed with a tachokomb. Hemostasis was performed, after which the dura mater was tightly sutured. The wound was sutured layer by layer and an aseptic dressing was applied.

At the end of the operation, when reflexes and adequate independent breathing were restored, the animals were transferred to independent breathing and extubated. In all cases, no significant episodes of hypotension or hypoxia were noted, and blood loss was up to 50 mL and was considered insignificant. In the postoperative period, all animals received antibiotic therapy (Ceftriaxone, 50 mg/kg in a 0.5% solution of novocaine, intramuscularly, once a day). Pain was minimized by administering Ketonal (15 mg/kg) intramuscularly to the monkeys on the first day after surgery, followed by 10 mg/kg of Ketonal on the second and third days after surgery.

### 2.5. Electrophysiology Assessment

Intraoperative monitoring of motor and sensory fibers for the hind limbs of animals was performed throughout the surgical procedure by recording transcranial motor-evoked potentials (MEPs) and somatosensory-evoked potentials (SSEPs) using the Neuro-IOM Neurosoft system (Neurosoft, Ivanovo, Russia; Neuro-IOM.NET software, version 2.0, https://neurosoft.com (accessed on 7 January 2026)), with recording of indicators before and after the moment of injury.

The latency (primary positive/negative deviation) and amplitude (maximum peak-to-peak value) parameters of the muscle response were assessed for the m. abductor hallucis and m. abductor pollicis brevis on both sides, with an active electrode placed in the motor point area. The bandwidth was set at 10–1000 Hz. An assessment was also made of the percentage of the initial latency and amplitude values. Stimulation of the motor area was performed using a pair of needle electrodes at points corresponding to the C1–C2/C2–C1 leads according to the international EEG electrode placement system “10–20%.” The grounding electrode was placed in the m. biceps brachii. Train stimulation was performed with 5 pulses, an interstimulus interval of 2 ms, a duration of 200 μs each, and a voltage of up to 500 V. For each response recording, at least 3 consecutive stimulations were performed to increase reliability.

The presence of a response is assessed, as were the amplitude and latency of the cortical SSEPs of the lower extremities in the form of the first positive (P1) and negative (N1) peaks. Alternate stimulation of the medianus and tibialis nerves on both sides in the area of the wrist joint and medial malleolus was performed using a pair of monopolar needle electrodes (cathode proximal) by applying direct current pulses, with a duration of 200 μs, a frequency of 3.12 to 4.12 Hz, and an amplitude from 4 to 10 mA (supramaximal amplitude was selected until a stable response was obtained). To obtain a single SSEP result, an average of 50 to 250 stimuli were used (depending on the severity of the SSEPs). The SSEP response was recorded using a pair of needle electrodes from the surface of the head with Cz-Fz lead (“10–20%”).

Electromyographic signals were recorded using bipolar electrodes implanted in the BIC, TRIC, FLE, EXT, RF, BF, TA, and GM. Electrophysiological recordings were performed during surgery to verify the spatial selectivity of the spinal implant and to precisely adjust its anatomical position. The delivery of a single current pulse (pulse duration 250 μs) 1.2–1.4 times higher than the motor threshold through the selected electrode contact caused motor responses in the leg muscles. EES-evoked responses included monosynaptic and polysynaptic components. EES was performed using a stationary implantable Precision Spectra generator (Boston Scientific, Marlborough, MA, USA) and three rod electrodes placed at the cervical and lumbar levels as described above. EES-evoked responses at the cervical and lumbar levels were recorded using IDinstruments (Austin, TX, USA) hardware and software and subcutaneous electrodes placed bipolarly above the motor point of the muscle. To determine the intensity of the stimulus required to elicit minimal limb muscle contractions (threshold) of responses, EES was performed with a duration of 250 μs, a frequency of 2 Hz, and a stimulus strength that varied in the range from 1 to 14 mA in 0.1 mA increments. EES-evoked motor response latencies were calculated as the time from stimulus pulse to the onset of the motor response of each muscle.

For the purposes of this study, EMG analysis was conducted on recordings obtained during EES-OFF periods to evaluate the baseline performance and stability of the implanted electrode platform. Therefore, specific stimulation artifact removal algorithms were not required. EMG and EES-evoked response data were collected at a sampling rate of 4 kHz (PowerLab; ADInstruments, Austin, TX, USA) and analyzed using custom code written in Object Pascal, Free Pascal Compiler (v.2) for Microsoft Windows. Notch (50 Hz) and bandpass (20–1000 Hz) filters were applied to EMG recordings to reduce environmental artifacts.

### 2.6. Kinematic Assessment

Within the framework of the platform we developed, we performed locomotion analysis—in an intact state, after electrode implantation (simultaneously with the recording of motor responses during walking) and during movement after spinal cord hemisection. The kinematic analysis consisted of assessing the range of motion in the joints while moving on a treadmill on four limbs with the head fixed using a special collar attached to the treadmill frame (see Supplementary Video S1). Fresh fruit (small pieces of apples, bananas, grapes) was offered to the animal as a reward during locomotion on the treadmill.

Gait registration was performed by filming the animal from two sides (left/right) followed by instrumental analysis of the angles in the limb joints using free Kinovea software (version 0.9.5, https://www.kinovea.org accessed on 7 January 2026) by marking video frames. Based on tables containing frame-by-frame angle values for a time interval during which the research object took several steps, the volumes of angular movement for three leg joints (hip, knee, and ankle) and two front limb joints (elbow and shoulder) were calculated using our original NeuroKin software (Certificate of registration No. for the computer program: RU 2024692126, 26 December 2024, https://www.fips.ru/registers-doc-view/fips_servlet (accessed on 7 January 2026)). The range of angular motion in the joints was calculated as the difference between the maximum and minimum angles in the joint during the step phase, based on the analysis of intervals including approximately 1.5 steps (intervals known to be longer than the duration of one step, automation algorithm). Then, the average values for several steps were calculated for a standard recording interval of about 1 min.

### 2.7. Statistical Analysis

Data on EMG and EES-evoked potentials are presented as the mean ± standard error of the mean (M ± sem). For each animal, latency values of EES-evoked potentials were averaged across five consecutive stimuli. Kinematic analysis, SEMP, and MEP data are presented as the median and interquartile range (IQR). The Shapiro–Wilk method was used to test for normal distribution of data. To determine statistical significance between paired conditions, a paired-samples *t*-test was employed. Statistical significance was set at (*p* < 0.05). All analyses were performed using SigmaPlot 11.0 (Systat Software, Inc., San Jose, CA, USA; https://systatsoftware.com accessed on 7 January 2026).

## 3. Results

We developed a platform for studying the neurophysiological mechanisms of neuromodulation in the treatment of spinal cord injury in non-human primates using a minimally invasive spinal stimulation and electromyographic signal recording system. This is a hybrid platform combining fully implanted EMG electrodes with wired head plugs, allowing the animal to perform full-amplitude and fast movements on a treadmill without tension and unfastening of the wires, as well as contactless adjustment of stimulation parameters. The neurostimulator operates using wireless recharging for long-term studies. It allows local stimulation of both the cervical and lumbar thickening of the primate spinal cord, and provides simultaneous recording of EMG signals from both the front and rear limbs. It is a biocompatible and well-tolerated complex which can be used for several months. This platform can be combined with various therapy options in the future, such as neural progenitor (or others) cells transplantation, implantation of various biocompatible scaffolds, and gene therapy. In our work, the developed head plug functioned successfully throughout the testing phase, and the osteoinductive material box ensured reliable fixation to the skull vault. Ultimately, the development of our proposed research platform allowed us to successfully conduct electromyographic and kinematic testing of three animals before and after spinal cord injury modeling using spinal cord electrical stimulation (Figure 1).

### 3.1. Recording of the Electromyographic Signal

**Figure 2:**
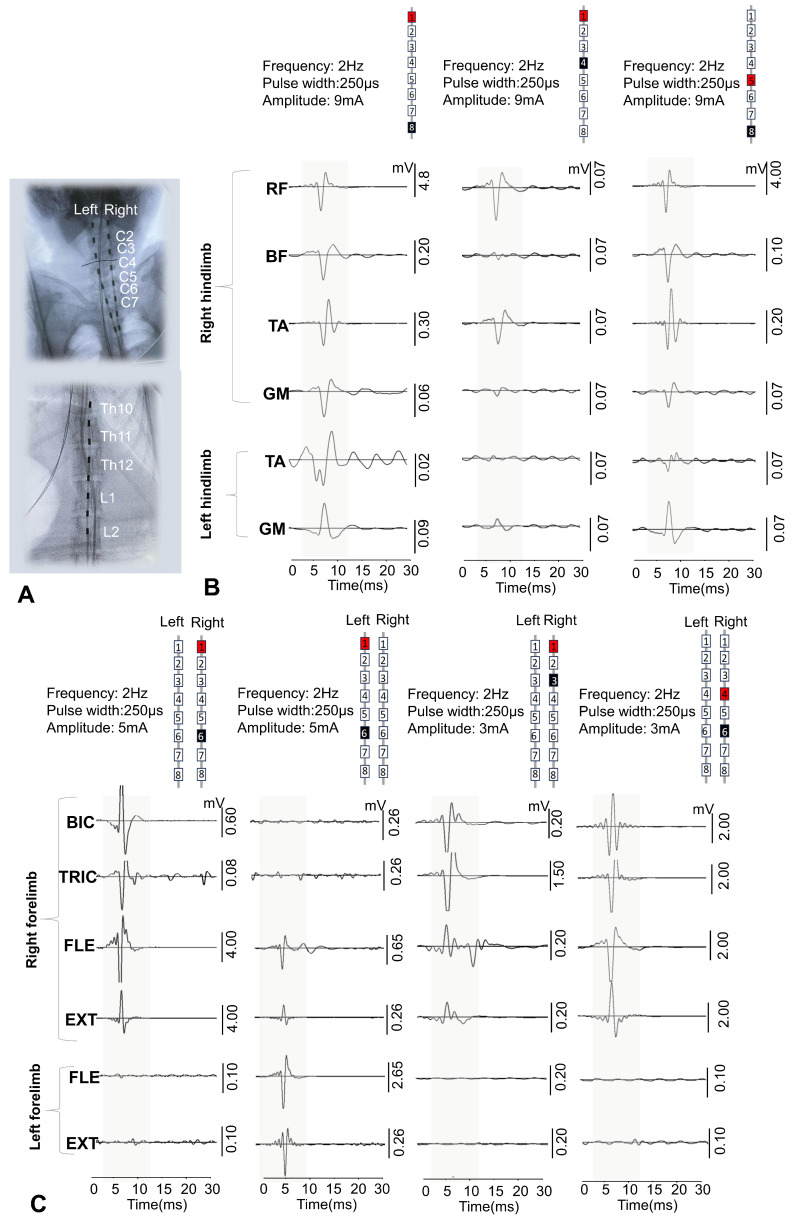
Assessment of evoked potentials of the muscles of the front and hind limbs caused by electrical stimulation of the spinal cord at the level of the cervical and lumbar thickening. (**A**) X-ray image of the position of the electrodes after implantation in one of the primates. (**B**) Example of recording EES-evoked motor responses of the muscles of the hindlimbs at different levels of stimulation. (**C**) Example of recording EES-evoked motor responses of the muscles of the front limbs at different levels of stimulation. The area of the evoked response is highlighted in gray. BIC—m. biceps brachii, TRIC—m. triceps brachii, FLE—m. flexor digitorum superficialis, EXT—m extensor digitorum superficialis, RF—m. rectus femoris, BF—m. biceps femoris, TA—m. tibialis anterior, GM—m. gastrocnemius medialis. In the schematic representation of the epidural electrodes, the numbers indicate the contact numbers, while the colors represent the configurations EES. Red and black squares indicate the anode and cathode, respectively.

The final positioning of the electrodes was confirmed using fluoroscopy and electrophysiological examination, so that the contact matrix covered all pools of motor neurons in the limbs (Figure 2A,B). Figure 2A shows examples of a control radiograph of the electrode position at the level of the cervical and lumbar thickening in one of the animals. Two epidural electrodes were placed parallel to each other at the level of the cervical spinal cord (in the DREZ areas), and the lumbar epidural electrode was located along the midline at the level of segments L3–S1. Stimulation with single pulses (2 Hz) during EES caused motor responses in anesthetized animals—contractions of the back muscles and front and hind limbs were observed. Figure 2B shows examples of recordings of evoked responses of BIC, TRIC, FLE, EXT, RF, BF, TA, and GM in response to electrical stimulation at the level of the lumbar and cervical segments of the spinal cord. Recording electrodes for electromyography implanted under the skin allowed the signal from the muscles to be recorded in all animals against the background of spinal cord stimulation. When the stimulus intensity necessary to elicit minimal contraction of the limb muscles was reached, an EES-evoked motor response with a latency period of approximately 3–6 ms was recorded. The thresholds for EES-evoked motor responses ranged from 2.5 to 4 mA.

Monitoring of intraoperative responses evoked by spinal cord stimulation allowed the epidural electrodes to be placed so that all the muscles under investigation were activated and selective activation of spinal sensorimotor networks was achieved. It was shown that rostral configurations predominantly activated proximal muscles, while caudal configurations activated distal muscles (Figure 2).

### 3.2. Electromyography Was Recorded in Freely Moving Monkeys

**Figure 3:**
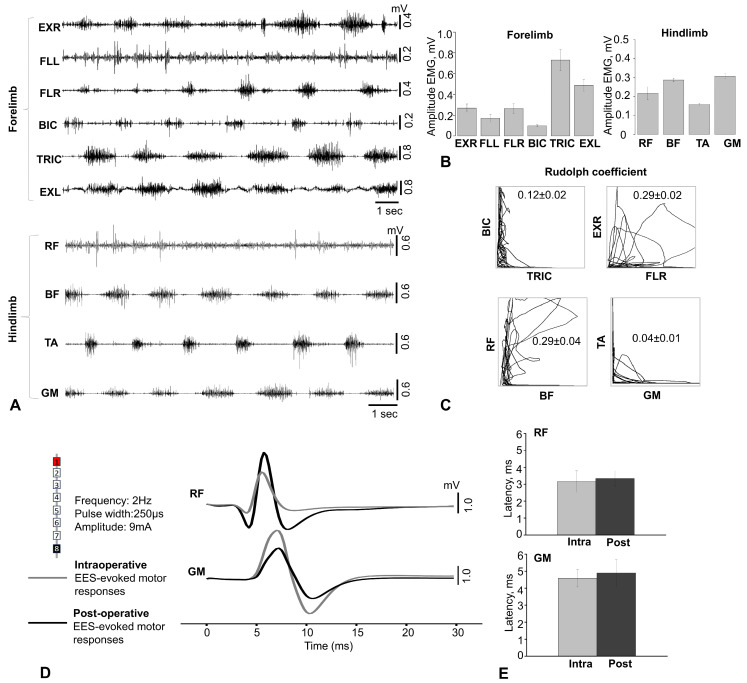
Electromyographic activity and EES-evoked potential characteristics during epidural electrical stimulation in rhesus macaques. (**A**) Example of EMG recording of the front and rear limbs during movement on a treadmill in an intact rhesus macaque 4 weeks after electrode implantation. BIC—m. biceps brachii, TRIC—m. triceps brachii, FLL—m. flexor digitorum superficialis left, FLR—m. flexor digitorum superficialis, RF—m. rectus femoris, BF—m. biceps femoris, TA—m. tibialis anterior, GM—m. gastrocnemius medialis. (**B**) Average values of EMG amplitude characteristics of the muscles of the anterior and posterior limbs of a primate while walking on a treadmill. (**C**) Analysis of antagonist muscle coordination using the Rudolf coefficient. A Rudolf coefficient close to 0 indicates mutually opposite skeletal muscle activity, while a coefficient close to 1 indicates coactivation (synchronous muscle contraction) of antagonists. (**D**) Examples of RF and GM muscle responses recorded during intraoperative and postoperative testing, with electrode configuration for EES used intraoperatively and postoperatively; In the schematic representation of the epidural electrodes, the numbers indicate the contact number, while the colors represent the EES configuration: black = cathode, red = anode. Each curve represents the average of five evoked responses. (**E**) Latency of evoked responses in RF and GM in primates. Data are expressed as mean ± SEM (n = 3). EES—epidural electrical stimulation.

Figure 3A shows an example of activation patterns recorded 4 weeks after implantation from ten limb muscles of an intact primate during movement on a treadmill at a speed of 0.9–1.1 km/h. The examples of activity shown in the figure demonstrate cyclic modulation patterns and are consistent with those expected for implanted muscles. Figure 3B shows the averaged EMG amplitude values for several cycles of movement on the treadmill, which also demonstrates good activation of these muscles. The average EMG amplitude characteristics of the forelimb muscles were 269.00 ± 39.00 μV for EXR, 172.00 ± 37.50 μV for FLL, 266.10 ± 47.30 μV for FLR, 97.90 ± 8.90 μV for BIC, 730.50 ± 101.70 μV for TRIC, and 487.70 ± 59.60 μV for EXL (n = 5) (Figure 3B). Figure 3C shows the coordination profile in antagonist muscles (BIC vs. TRIC, EXR vs. FLR, TA vs. GM, BF vs. RF) during movement. Analysis of muscle activation based on the Rudolf coefficient (L-shaped graph) showed that muscle coordination for the proximal muscles of the forelimbs averaged 0.12 ± 0.02, and for the distal muscles, 0.29 ± 0.02. For the proximal muscles of the hind limbs, it averaged 0.29 ± 0.04, and for the distal muscles, it averaged 0.04 ± 0.01 (Figure 3C). A coefficient value close to 0 and an L-shaped graph indicate a reciprocal activation profile of antagonist muscles. Overall, these results show that the proposed model of an implanted system for recording EMG signals in non-human primates allows for successful measurement of chronic EMG activity 4 weeks after implantation with high quality during movement on a treadmill.

Figure 3D shows an example of EES-evoked muscle responses at the level of the cervical and lumbar thickening 4 weeks after implantation of epidural and EMG electrodes. EES-evoked responses were recorded at suprathreshold intensity (9 mA). EES-evoked motor responses were generally similar in shape and number of peaks between intra- and postoperative recordings, but the latency period of responses from the proximal (RF) and distal (GM) muscles of the hind limb (RF = 3.16 ± 0.36 and 3.34 ± 0.26 ms (n = 3) and GM = 4.58 ± 0.29 and 4.90 ± 0.45 ms (n = 3) for the intra- and postoperative periods, respectively) were statistically insignificant (RF, *p* = 0.774; GM, *p* = 0.700; Figure 3E). It should be noted that although three animals maintained good activity in at least ten muscles, only six muscle electrodes remained viable in the first animal included in the study. This failure was due to the animal opening the wound on its right hind limb and pulling out the implanted electrodes.

Thus, after 4 weeks, there was no displacement of the epidural electrodes and no deterioration in the quality and quantity of electromyographic signals.

### 3.3. Assessment of Evoked Potentials After Spinal Cord Injury

**Figure 4:**
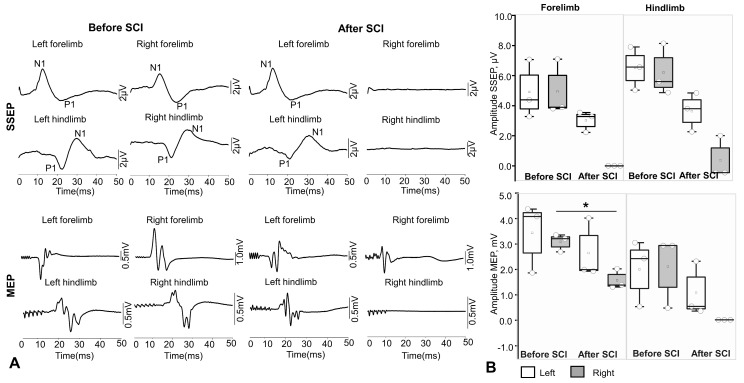
Assessment of motor- and sensory-evoked potentials in one of the rhesus macaques after spinal cord injury at the level of the C3–C4 vertebrae. (**A**) Examples of recording somatosensory and motor-evoked potentials of the front and hind limbs of a rhesus macaque. (**B**) Average amplitude values of evoked potentials in rhesus macaques (n = 3) before and after modeling spinal cord injury at the C3 –C4 vertebrae level. Gray circles—amplitude values of evoked responses for each animal. Data for SSEP and MEP amplitudes are presented as the median (horizontal black line inside) with interquartile range. * *p* < 0.05, paired *t*-test.

Five weeks after implantation of chronic EES and EMG electrodes, spinal injury was simulated in rhesus macaques. All animals (n = 3) underwent right-sided hemisection and exhibited ipsilateral (right-sided) hemiparesis, most pronounced in the distal limbs. In the first few days after injury, the animals were sedated to reduce stress. The animals then gradually began to move around the cage, using various elements of the enriched environment for support. In all animals, before and after lateral hemisection, intraoperative monitoring of SSEPs and MEPs of the front and hind limbs was performed to control the localization and extent of spinal cord damage. Figure 4A shows examples of SSEP recordings from one of the animals during stimulation of the tibialis and median nerves and MEP recordings from the front (abductor pollicis brevis muscle) and rear (abductor hallucis muscle) limbs of a rhesus macaque during spinal injury simulation. After injury, no responses were detected from the right front and hind limbs: cortical SSEPs were not recorded in two of three animals (a response was observed from the right lower limb, which was significantly reduced) according to the response criterion (A_pp > 3 × SD of noise in the 0–5 ms window). Visual assessment also did not reveal reproducible components. The latency of cortical SSEPs was assessed as follows: for the upper limbs: P1 17.54 ± 1.924 ms, N1—10.91 ± 1.13 ms; for the lower limbs: P1 21.60 ± 1.68 ms, N1—31.37 ± 0.53 ms. The latency of MEPs of the anterior limb muscles averaged 9.48 ± 0.40 ms, and in the lower limbs, 16.98 ± 0.67 ms. Figure 4B shows the averaged amplitude data of evoked responses before and after spinal cord injury in primates. Thus, the amplitude of P1–N1 cortical SSEPs on the left before injury averaged 6.70 [5.23 ± 7.98] µV, and after SCI, it was 5.78 [5.08 ± 8.20]. The amplitude of P1–N1 cortical SSEPs on the right before injury averaged 4.06 [2.59 ± 5.06] µV, and after SCI, it was not recorded. The amplitude of P1–N1 cortical SSEPs on the left before injury for the hind limbs (n. tibialis) averaged 4.39 [3.2, 7.07] µV, and after SCI, 2.22 [1.07, 3.54]. The amplitude of P1–N1 cortical SSEPs on the right before injury averaged 3.9 [3.80 ± 7.1] µV for the front limbs, and was not recorded after SCI. The amplitude of MEP in the left hind limb before spinal cord injury was 2410.00 [520.66, 3033.33] μV, and after SCI, 531.33 [344.33, 2301.00] μV; in the right hind limb before spinal cord injury, the MEP amplitude was 2676.66 [462.00, 2920.00] μV, and after injury, it was not recorded. The MEP amplitude in the left front limb before spinal cord injury was 4080.00 [1863.33, 4373.33] μV, and after SCI, 1993.00 [1930.00, 4020.00] μV; in the right front limb before spinal cord injury, the MEP amplitude was 3206.66 [2690.00, 3353.33] μV, and after injury, the values decreased statistically significantly to 1390.00 [1306.66, 2023.33] μV (n = 3, *p* = 0.010) (Figure 4B).

Thus, the method of hemisection in non-human primates proposed in this study allows effective modeling of spinal cord injury at the level of the right cervical thickening under intraoperative control.

### 3.4. Assessment of Changes in the Kinematic Characteristics of Primate Locomotion After Spinal Injury Simulation

**Figure 5:**
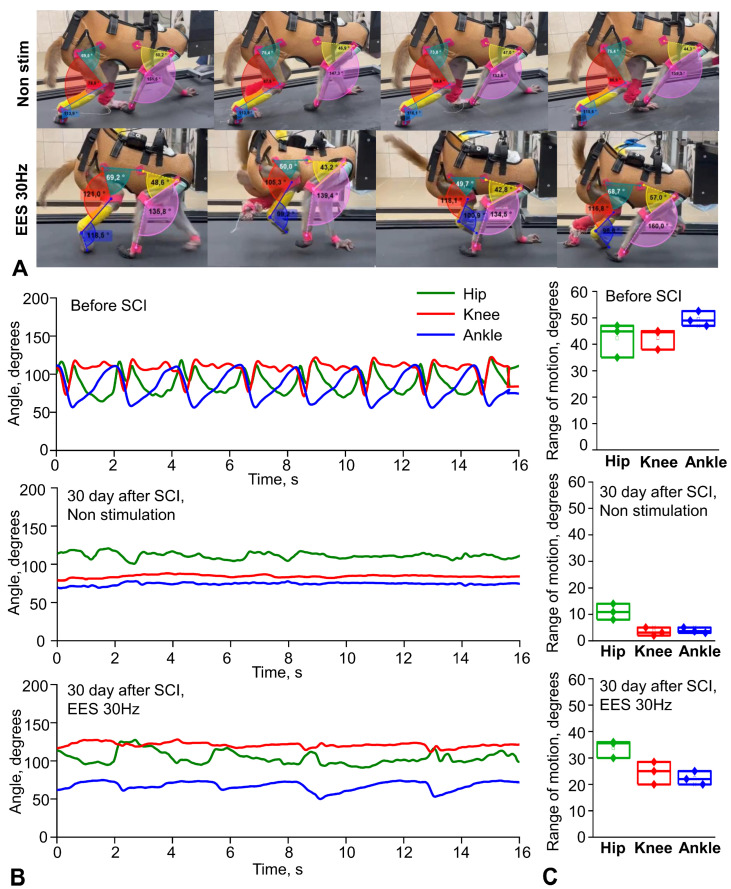
Assessment of the kinematics of movements during walking on a treadmill in rhesus macaques before SCI and 30 days after spinal injury modeling. (**A**) Schematic illustration of the recording of the volume of angular movement in the joints of the front and hind limbs of an animal on a treadmill during free movement (without stimulation and during neuromodulation). Original screenshots from Kinovea software. (**B**) Representative goniograms of three joints of the right hindlimb in one individual animal. (**C**) Mean range of motion in right hindlimb joints during treadmill locomotion before SCI, after SCI (30 day), and during epidural electrical stimulation (EES). Data are presented as the median (horizontal black line inside) with interquartile range (n = 3).

After simulating spinal injury, the kinematic characteristics of rhesus macaque locomotion were assessed. Figure 5A shows examples of changes in angular range of motion during treadmill walking in rhesus macaques without spinal cord electrical stimulation and during stimulation at the level of the lumbar thickening.

The analysis revealed that the average angular motion volumes in the three joints of the right limb of the rhesus macaque walking on a treadmill decreased sharply after spinal cord injury (Figure 5B, see Supplementary Video S2). The volumes of angular movement in the joints of the right hind limb during locomotion on a treadmill decreased in the hip joint to 10.9 [8.7; 13.2] degrees (n = 3), in the knee joint—to 3.1 [2.3; 4.5] degrees (n = 3), and in the ankle joint—to 3.6 [3.1; 4.6] degrees (n = 3). The angles of movement in the joints of the right front limb during locomotion on a treadmill decreased in the elbow joint to 11.0 [8.7; 16.7] degrees (n = 3) and in the shoulder joint to 4.0 [3.2; 5.2] degrees (n = 3). At the same time, during complex therapy with the use of neuromodulation at the level of the lumbar thickening of the spinal cord (frequency of 30 Hz, duration of 250 μs, intensity of 1–12 mA), an increase in the volume of angular movement in the joints of the hind limbs was found (Figure 5B). The volumes of angular movement in the joints of the right hind limb during locomotion on a treadmill with this stimulation were increased in the hip joint to 35.6 [31.4; 35.9] degrees (n = 3), in the knee joint up to 25.0 [21.2; 27.6] degrees (n = 3), and in the ankle joint up to 22.0 [20.5; 24.2] degrees (n = 3). The volumes of angular movement in the joints of the right front limb during locomotion on the treadmill were 55.0 [47.5; 58.5] degrees (n = 3) in the elbow joint and 49.0 [47.5; 52.45] degrees (n = 3) in the shoulder joint. Changes in the kinematics of the left hindlimb were also recorded using video cameras, markers, and numerical analysis, but they were similar to the period before SCI, which was confirmed by video recording of the symmetry of movements with a camera.

## 4. Discussion

Our study showed that minimally invasive implantation of epidural electrodes with a wireless stimulator, along with implantation of invasive myographic electrodes followed by hemisection of the spinal cord at the cervical level, is an ethically acceptable platform for studying the neurophysiological mechanisms of neuromodulation in the treatment of SCI in NHP. This platform is easy to implement, allows for the assessment of kinematics and myography in motion, and can be combined with various treatment options, such as neural progenitor cell transplantation, implantation of various biocompatible scaffolds, gene therapy using AAV, implantation of cortical grids, development of BCI, etc.

More than twenty years of preclinical and clinical research has shown that EES at the lumbosacral level of the spinal cord can promote the activation of spinal locomotor circuits after SCI [16,17,31,32]. Using computer modeling [33,34,35] and experimental studies conducted on animal models and humans with spinal cord injury [36,37], it has been convincingly proven that electrical stimulation of the spinal cord at the lumbosacral level primarily involves large myelinated afferent fibers passing through the dorsal roots of the spinal cord [38]. Spinal cord electrical stimulation thus not only activates the spinal cord networks responsible for locomotion through interneurons and motor neurons in the lumbar thickening [39], but also modulate the spinal cord network in people after SCI [40]. These studies highlight the need to develop the potential of stimulation technologies using EES to transform SCI medicine. In our study, we performed electrical stimulation in a non-contact manner using implanted permanent generators, but the responses were recorded in a contact manner using a patented wired head plug.

The use of a minimally invasive rod electrode for EES instead of suturing a wired electrode to the dura mater significantly reduces surgery time, as minimally invasive placement does not require laminectomy. Intraoperative positioning and selection of optimal electrode configurations are of paramount importance [26]. Previously, animal models have successfully demonstrated that the localization of epidural electrodes relative to the posterior roots plays a decisive role in determining the configuration and intensity of stimulation required to activate selected spinal cord circuits [41]. Intraoperative monitoring of electrophysiological parameters during surgery allowed for precise positioning of the stimulating electrodes. EES applied at different levels of the spinal cord using specific electrode configurations allowed for selective activation of different pools of motor neurons in the spinal cord. In rhesus macaques, rostral configurations predominantly activated proximal muscles, while caudal configurations activated distal muscles (Figure 2). In addition, the use of wider configurations for EES (one and eight contacts on rod electrodes) along the midline at the level of the lumbar thickening of the spinal cord probably allowed for the activation of a greater number of spinal cord segments, resulting in a greater number of muscles being involved in the response and a higher amplitude of EES-evoked responses (Figure 2). Earlier studies have shown that stimulation of these areas activates the L1–S1 spinal cord segments in humans [18,42]. These results indicate that the use of a rod electrode in EES in non-human primate models can successfully activate the most proximal and distal muscles of the leg, as well as activate muscles on only one side. However, in the early postoperative period, there is a risk of electrode tip migration when using a rod electrode, since with this technology, only the distal part of the electrode is fixed at its exit point [43]. Our experience shows that when two cervical electrodes were implanted using the technique we developed—in the DREZ zone on the right and left—there was no electrode migration in any case. The problem of electrode migration may arise for the central lumbar electrode in the first two weeks after implantation. In cases of significant electrode migration detected by electromyography or CT, the electrode can be easily repositioned (minimally invasive electrode repositioning takes no more than 30 min). After three weeks, a connective tissue capsule forms around the electrode, making migration impossible. Thus, from our point of view, minimally invasive installation of an 8-channel rod electrode has many more advantages than disadvantages compared to open installation. In the future, with the development of this technology at NHPs, it may be possible to create flat custom electrodes for animals, also with minimally invasive installation. Such custom electrodes have already demonstrated their advantages in patients [44].

It remains debatable whether it is possible to completely abandon wired signal transmission at the current stage of neurotechnology development. On the one hand, it is quite obvious that wireless epidural electrical stimulation and muscle response recording are preferable in the case of non-human primates. The Courtine G. group implemented the idea of wireless transmission of cortical signals from the motor cortex to spinal efferents via a brain–computer interface. This idea was investigated in an experiment on non-human primates [32], and was also implemented in a pilot study involving patients [45].

These studies used a wireless system: six antennas and a receiver were used to transmit 25 broadband neural signals (bandwidth from 0.1 Hz to 7.8 kHz, sampling rate of 22 kHz), customized in collaboration with Medtronic. In addition to being quite expensive and requiring constant recharging (which, in the case of primates, is almost as difficult to implement as a wired connection and usually requires sedation), its use in broadband transmission mode is accompanied by heating, which can be unsafe when transmitted to patients. Unlike wireless transmission, wired transmission does not generate heat at high flow rates and high frequencies of transmitted signals, does not require recharging, and, when used with a treadmill that secures the animal with a special collar, does not require sedation for connection. In any case, from our point of view, two transmission methods (wireless and wired) are preferable to one, so on our platform, we performed wireless epidural stimulation and wired recording of myographic signals with 4 kHz sampling.

The complexity of postoperative care and ethical and regulatory issues impose significant limitations on the choice of SCI model for non-human primates [28]. To test our experimental platform for studying the neurophysiological mechanisms of neuromodulation in spinal injury therapy, we adopted a cervical lateral hemisection model [26]. This SCI model does not result in spontaneous recovery [46,47]. In our study, all animals showed no signs of motor recovery on the side of spinal cord injury for 5 weeks after spinal injury modeling.

Training and implanting anything in NHPs is technically a very difficult experimental task, requiring an extremely refined methodology due to the behavioral characteristics of primates. In total, 20 Rhesus macaques participated in our project for the successful completion of all procedures for developing kinematic skills, training, implantation of electrodes, generators, and head plugs, followed by data recording. The most important factor for the successful implementation of the project was the selection of compliant animals that were easy to train in quadrupedal walking on a treadmill. Our experience shows that among animals kept in enclosures, captured and kept for 3 months in individual cages with an enriched environment, no more than 25% begin to walk independently on the treadmill. Animals should be no more than 2–2.5 years old at the start of treadmill training, weigh up to 5 kg, and be kept in individual cages for at least three months (the optimal period is six months: three months in an individual cage and three months with a collar for connection to the treadmill).

As a result of this initial testing, we selected five animals out of 20 that showed stable quadrupedal walking on the treadmill. After installing the electrodes, we had to exclude two animals during the experiment. The first one had the initial version of the head plug installed, and it was attached to the head using a photo-curing composite. However, this proved insufficient to withstand the high mechanical loads during the animal’s natural behavior, leading to detachment and subsequent breakage of the plug. Consequently, the EMG electrodes had to be removed to ensure animal safety. This specific failure mode was the primary catalyst for the development of our second-generation osteoinductive head plug (Patent RU 2 829 633 C1). By moving from a superficial composite bond to a design that promotes osseointegration, we achieved the necessary structural integrity to prevent such failures in subsequently used animals. In the second excluded animal, the stress-relief loops at the entrance to the head plug were located too superficially under the skin; as a result, despite pharmacological support with Gabapentin and Trazodone, the animal was able to remove the loops and tear off part of the electrodes. This was likely due to repetitive mechanical stress from the animal’s grooming behavior. Thus, we made changes to the second-generation head plug design to address these technological shortcomings: we designed a new plug that is 20% flatter (now 25 mm above the bone level) so that it protrudes less from the scalp and does not pose a risk of damage. Also, the cementless method of fixation to the skull uses a method that stimulates direct bone fusion with the implant (osteoinductive material), and the entire array of electrodes and connectors is concealed within a durable 3D-printed material housing. A shock-resistant cap was installed on the implanted camera. It completely blocks finger access to the wires and the wound edges (Figure 1). In all subsequent animals, the exit of the electrodes under the skin to the plug was additionally reinforced with a titanium plate fixed to the skull (see Supplementary Figure S2), and we provide X-ray verification of electrode positions after the surgery (see Supplementary Figures S3 and S4). Another characteristic complication during the installation of the wireless generator was skin trauma and the formation of bedsores on the skin in the projection of the generator. As a result of bedsores, the generator had to be relocated for two animals. To avoid this complication, vests were made for all animals to prevent traumatic contact between the skin above the generator and the hard elements of the cage.

## 5. Conclusions

In this proof-of-concept preclinical study, a multi-electrode platform was developed that allows wireless epidural electrical stimulation and wired electromyography in non-human primates while moving on a treadmill. The reproduced model of spinal cord hemisection at the cervical level allows for stable conduction loss in the ipsilateral half of the spinal cord and stable but ethically acceptable neurological deficit. The developed platform is applicable both for neurophysiological studies, including the development of control neurointerfaces, and for assessing the safety and efficacy of regenerative therapy and various combined approaches.

## 6. Limitations

The main limitation of this study is the small number of animals used (n = 3). This small sample size is due to the technical complexity of chronic electrode implantation procedures, modifications to the head implant, and the attrition of 75% of the total number of animals (n = 20) due to behavioral issues and difficulties in training (treadmill skills). Despite these limitations, this “proof-of-concept” study provides valuable preliminary data on a new, bioethically acceptable platform for spinal cord injury research using non-human primates. Future studies involving a larger number of animals are needed to verify our observations and refine the stimulation parameters. Also, while intraoperative physiological markers confirm the injury in our study, future studies would benefit from correlative histology to map the exact lesion boundaries and secondary degeneration.

## Data Availability

The raw data are available upon reasonable request.

## Supplementary Materials

The following supporting information can be downloaded at: https://www.mdpi.com/article/10.3390/biomedicines14010166/s1, Figure S1: Flowchart for animal selection; Figure S2: Head plug installation steps. (A) Primary fixation of a plate made of osteocompatible biomaterial using self-tapping screws and light-curing composite (primary fixation of plugs)—view after removal of the liner. (B) The prefinal stage—the head plug is fully installed; the wire harness is fixed with a titanium plate and two self-tapping screws. Figure S3: X-ray verification of electrode positions after the implantation. (A) Whole body pilot on CT scan. (B) Cervical electrode positions; (C) X-ray of generator position; Figure S4: Example pre- and post-operative electrode imaging. (A) Position of the electrodes after completion of the implantation procedure (prior to the spinal cord lesion). (B) Intraoperative inspection of the electrode implanted in the right DREZ area after dural incision (the electrode is indicated by the arrow). (C) Verification of the electrode positions after lesion induction and dural closure. Video S1: Example of an animal performing the training on the treadmill with kinematics analysis; Video S2: Example of an animal performing the training on the treadmill after SCI with epidural spinal cord stimulation at the lumbosacral level.

## Abbreviations

The following abbreviations are used in this manuscript:

BF	m. biceps femoris
BIC	m. biceps brachii
EI	electrode implantation
EMG	electromyography
EES	epidural electrical stimulation
EXT	m. extensor carpi ulnaris
FEP	fluorinated ethylene propylene
FLE	m. flexor digitorum superficialis
GM	m. gastrocnemius medialis
IQR	interquartile range
MEP	motor evoked potential
NHP	non-human primates
RF	m. rectus femoris
SC	spinal cord
SCI	spinal cord injury
SSEP	somatosensory evoked potential
TA	m. tibialis anterior
TRIC	m. triceps brachii
