# Supplementary figures and images for: Assessment of Chronic Multi-Electrode Spinal Cord Electrical Stimulation and Electromyography Platform in Non-Human Primates
Source: Biomedicines. 2026 Jan 13;14(1):166. doi: 10.3390/biomedicines14010166 (PMC12839459; doi:10.3390/biomedicines14010166)

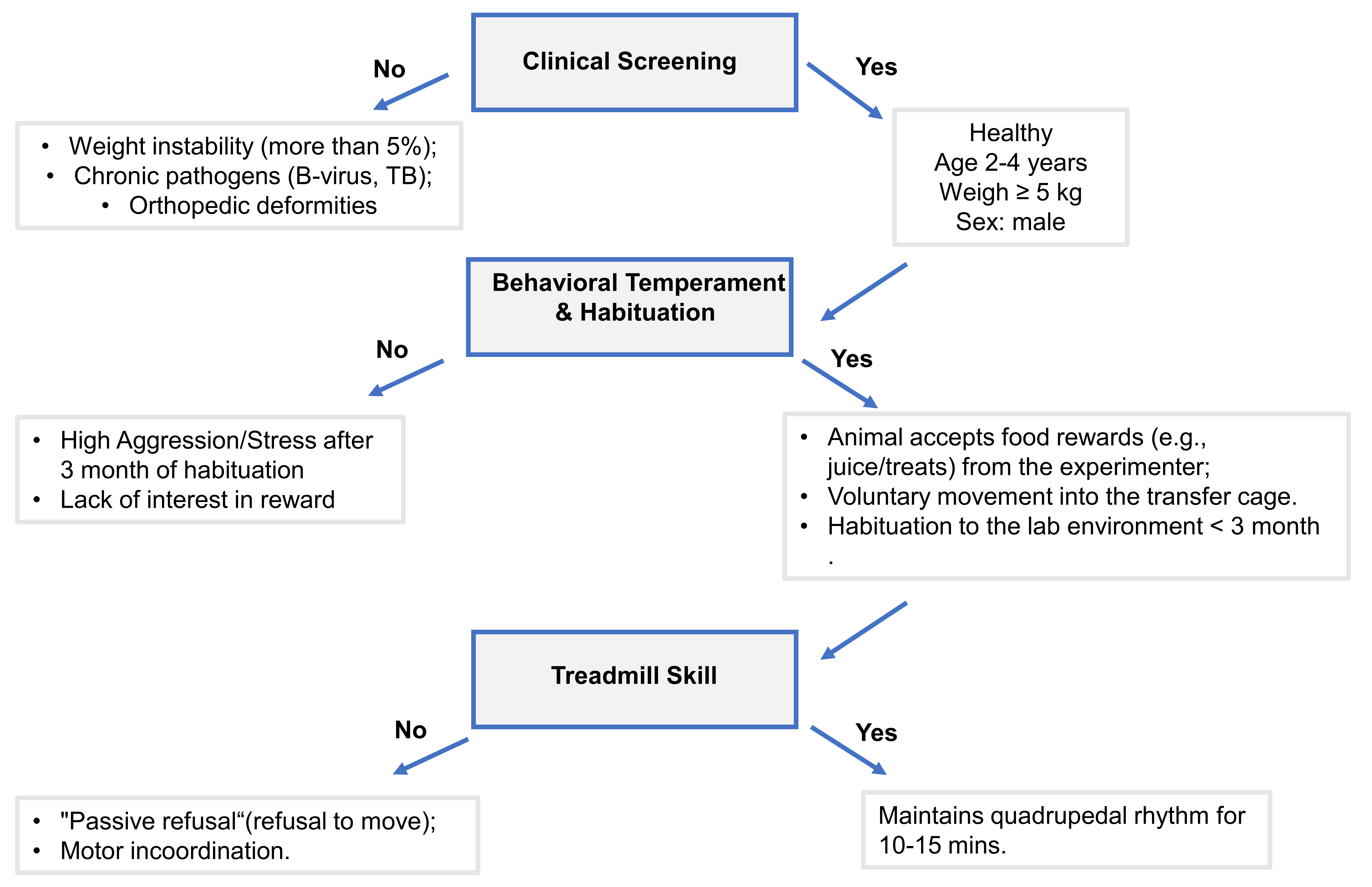

Supplement: Supplementary file 1 [file biomedicines-14-00166-s001.zip › Supl fig 1 2026.tif]

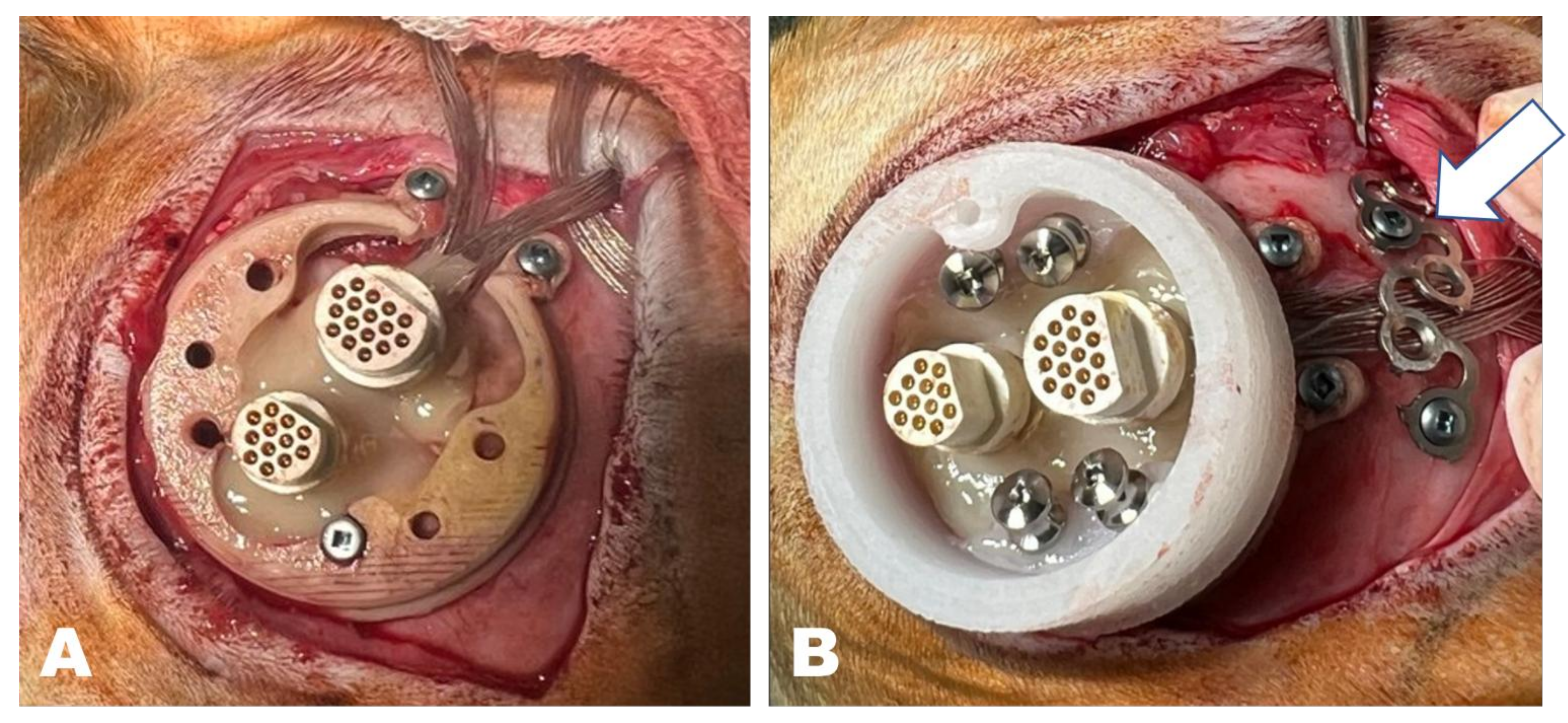

Supplement: Supplementary file 1 [file biomedicines-14-00166-s001.zip › Supl fig 2 2026.tif]

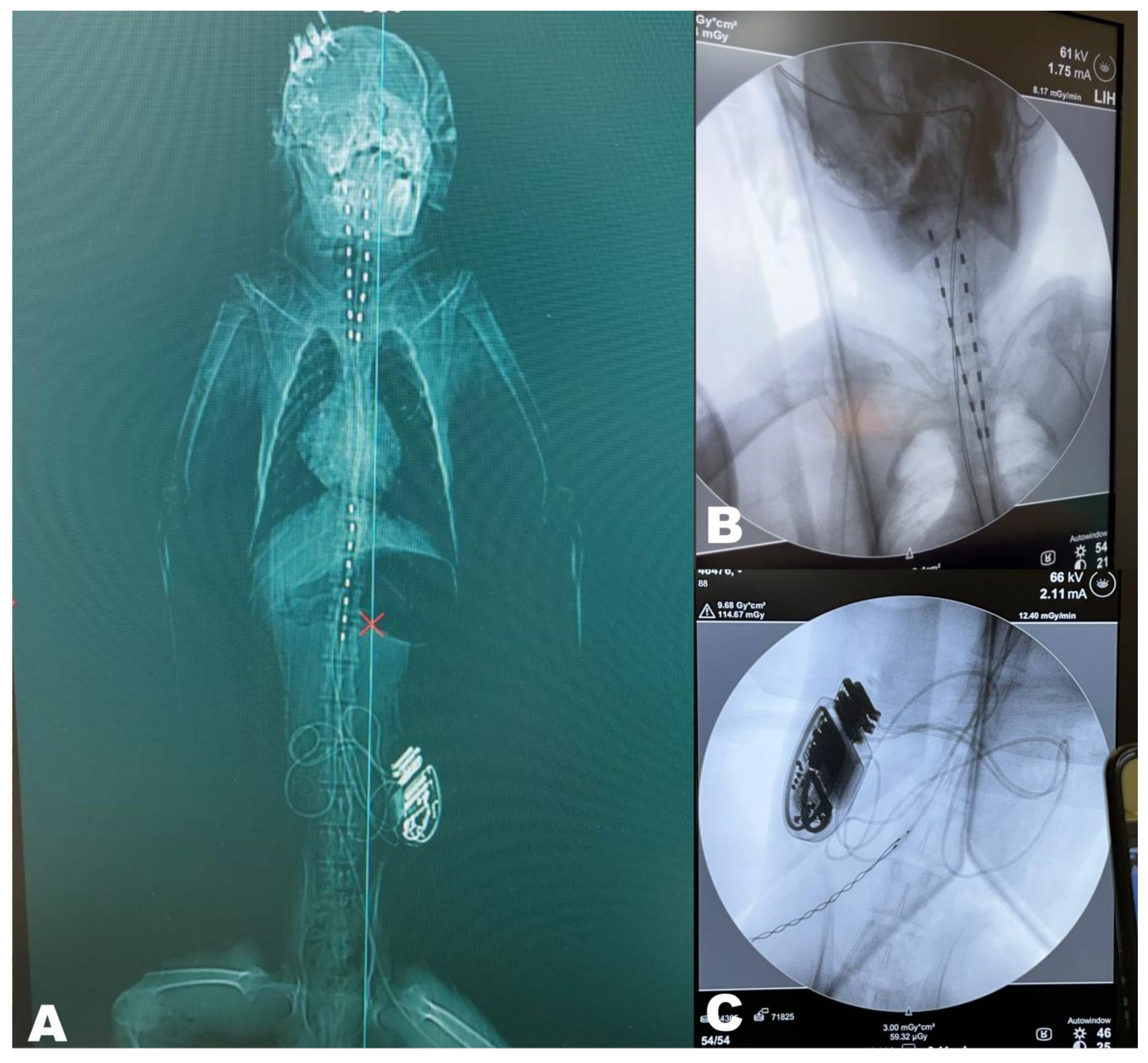

Supplement: Supplementary file 1 [file biomedicines-14-00166-s001.zip › Supl fig 3 2026.tif]

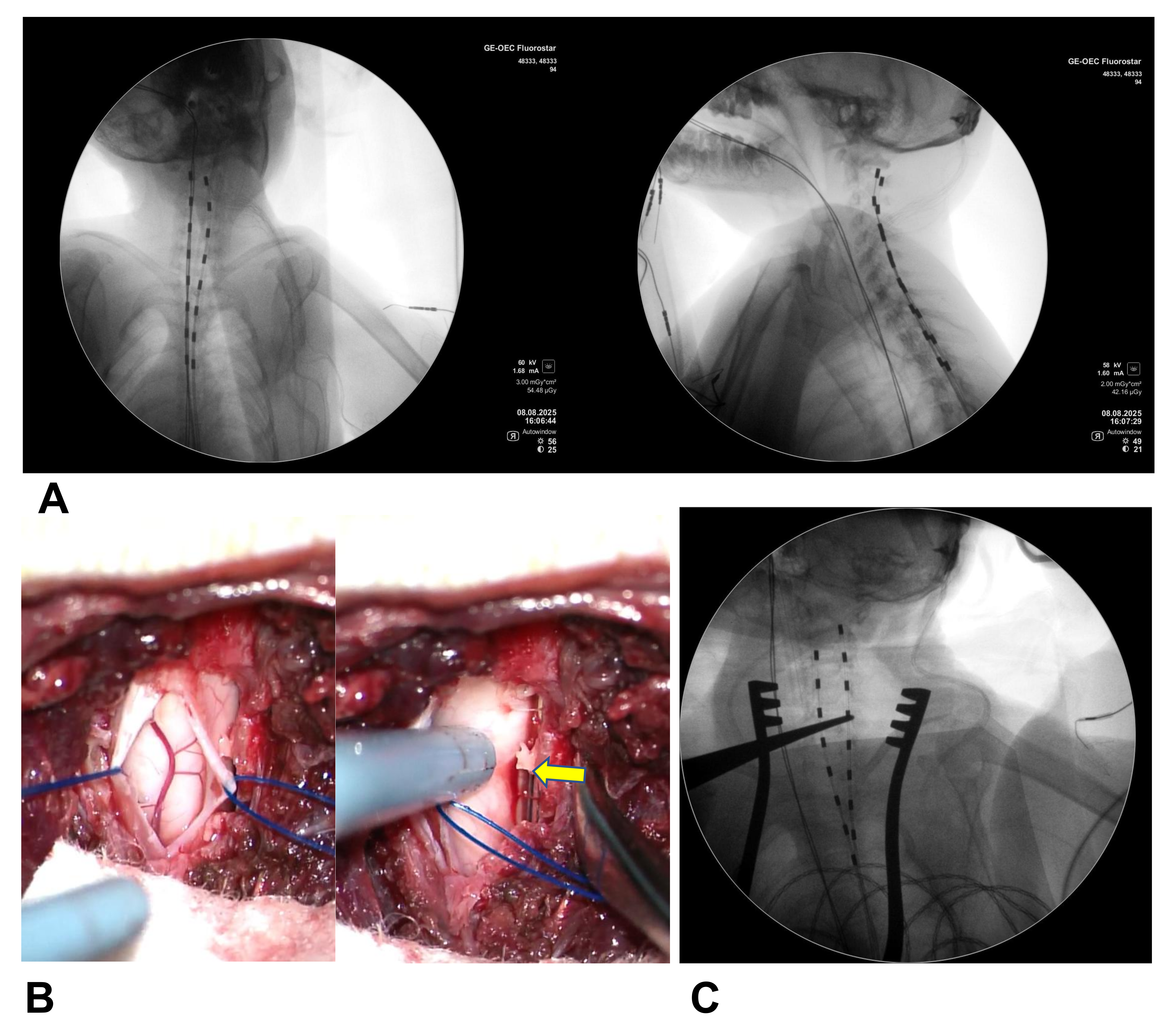

Supplement: Supplementary file 1 [file biomedicines-14-00166-s001.zip › Supl fig 4 2026.tif]
